# Impact of Optical Cavity on Refractive Index Sensitivity of Gold Nanohole Arrays

**DOI:** 10.3390/bios13121038

**Published:** 2023-12-18

**Authors:** Maria A. Shokova, Vladimir E. Bochenkov

**Affiliations:** Chemistry Department of Lomonosov, Moscow State University, 119991 Moscow, Russia; mariia.shokova@chemistry.msu.ru

**Keywords:** surface plasmon resonance, label-free sensing, sensitivity, FDTD simulations

## Abstract

Refractive index sensing based on surface plasmon resonance (SPR) is a highly efficient label-free technique for biomolecular detection. The performance of this method is defined by the dielectric properties of a sensing layer and its structure. Nanohole arrays in thin metal films provide good refractive index sensitivity but often suffer from a large resonance linewidth, which limits their broad practical application in biosensorics. Coupling the broad plasmon modes to sharp resonances can reduce the peak widths, but at the same time it can also degrade the sensitivity. Here, we use Finite-Difference Time Domain simulations to study the factors affecting the sensing performance of gold-silica-gold optical cavities with nanohole arrays in the dielectric and top metal layers. We demonstrate that by tuning resonator size and inter-hole spacing, the performance of the biosensor can be optimized and the figure of merit of the order of 5–7 is reached.

## 1. Introduction

Optical biosensors based on localized or propagating surface plasmon resonance have emerged as a powerful tool for a range of applications, including in healthcare, biochemistry, environmental monitoring, and food safety control [1,2,3]. Such sensors aim at providing efficient label-free detection of target biomolecules by responding to minute changes in the refractive index of the media near the surface of a plasmonic nanostructure. The performance of a refractive index sensor is determined by the geometric shape, size, symmetry, and spatial arrangement of the plasmonic units, which facilitates the active search for new nanostructures and ways to improve existing plasmonic sensor systems. To characterize the performance of a biosensor, two parameters are generally used: sensitivity *S* and figure of merit (*FOM*). The former is defined by the spectral shift of a resonance peak Δλ caused by the change of the refractive index Δn:

(1) $$S=\frac{\Delta \lambda }{\Delta n}.$$

while the latter is calculated as the ratio between the sensitivity *S* and the full width at half maximum FWHM of the resonance:

(2) $$FOM=\frac{S}{FWHM}.$$

Numerous plasmonic nanostructures, including dispersed or substrate-supported metal nanoparticles, 2D or 3D nanoparticle clusters, and metal films with periodic or aperiodic patterning, have been investigated so far as potential sensing elements of a biosensor [4,5,6]. Particularly interesting plasmonic systems for biosensing applications are the periodic and the short-range ordered nanohole arrays (NHAs) in thin metal films [7], since such nanostructures can be fabricated relatively easily on large-area substrates at cm${}^{2}$ scale using colloidal lithography [8,9,10]. The flexibility, low cost, and high throughput of fabrication make these nanostructures advantageous compared with many other systems.

Typically, NHAs demonstrate moderate bulk refractive index sensitivities of the order of 200–300 RIU/nm. A number of studies have investigated the factors that influence the sensitivity of NHA, such as the diameters of the nanoholes, their ordering, and even the incident angle of the illuminating light [7,11,12,13].

In recent studies, we have demonstrated that the refractive index sensitivity of gold NHAs can be significantly improved through the addition of a perforated silica layer or by etching the dielectric substrate under the gold film [14]. This eliminates the so-called “substrate effect”, which refers to the lower sensitivity observed in nanostructures with substrate support compared with those without. This effect occurs because a substantial portion of the enhanced electric field is obstructed within the substrate material with a higher refractive index, making it inaccessible to analyte molecules [15].

Further improvement in the performance of plasmonic NHA-based biosensors could be achieved by reducing the spectral width of a resonance to increase the *FOM* of a sensor material. Typical values of FWHM for short-range ordered gold NHAs are in the order of a few hundreds of nm, depending on the spectral position of the resonance. The width is defined by the radiation damping of the metal and the strong scattering of the surface plasmon wave by aperiodically arranged nanoapertures [16,17]. This results in very low values of a Q-factor, which limits the range of practical applications of such materials in sensorics.

The ordering of nanoholes can result in a 2–3 times reduction of FWHM [11]. Further, the resonance linewidth can be reduced by coupling plasmons to a system with a narrow resonance, such as photonic cavity, due to the modified photonic density of states. This effect has been demonstrated in various systems, including gold nanorods [18], nanodisks [19], and nanorings [20] positioned above a gold mirror. However, to the best of our knowledge, there is currently no data available on the impact of coupling in the NHAs–photonic cavity system on refractive index sensing performance. In this study, we employ finite-difference time-domain (FDTD) simulations to investigate the spectral properties and refractive index sensitivity of gold NHAs with and without an additional gold layer that forms an optical resonator. By carefully selecting the hole diameter and inter-hole spacing, as well as tuning the distance between the two gold layers, we enable the coupling of electromagnetic light modes with the plasmon modes of the NHAs. Our findings provide insights into how these modifications can enhance the performance of a substrate-supported plasmonic nanostructure.

## 2. Simulation Details

**Figure 1:**
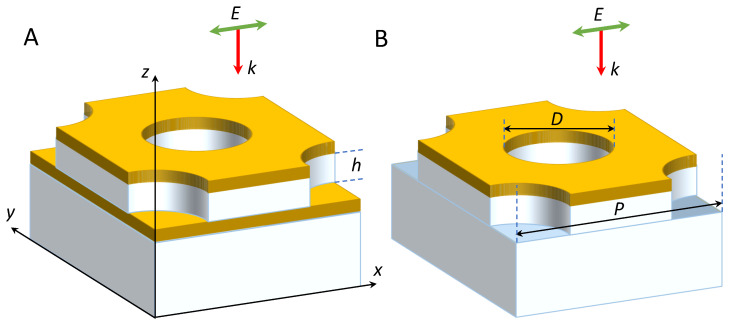
Schematic representation of the simulated structures (the unit cells): (**A**) the three-layer Au/SiO${}_{2}$/Au system with nanoholes in the top metal and dielectric layers; (**B**) the elevated Au/SiO${}_{2}$ NHA. Gold is represented by the yellow color, silica by the white color.

Numerical simulations are performed using the FDTD++ software (version 1.7). Optical properties of two system types are compared. The schematics of the simulated systems is demonstrated in Figure 1. The first one is a substrate-supported gold–silica–gold nanostructure in which the top metal and dielectric layers are pierced by cylindrical nanoholes of diameter *D*. The holes are arranged in a hexagonal 2D lattice with a period of *P*. Under the silica layer, there is a continuous gold layer. The nanostructures of the second type are identical, but do not contain the bottom gold layer. These systems represent the ‘elevated’ NHAs studied earlier [14]. To demonstrate the effect of changing the hole diameter, we consider systems with D=80, 100 and 120 nm. The thickness of the gold layers is 20 nm, while the thickness of the dielectric spacer *h* is varied from 20 to 200 nm.

The dielectric function of gold is represented by a fit to experimental data [21]. A constant refractive index of n = 1.52 is used for the silica layer and the substrate. Periodic boundary conditions apply in the *x* and *y* directions, and the perfectly matched layer is used to absorb electromagnetic waves propagating along the *z* axis. In the region of the upper perforated layer of gold, a constant size mesh of 3×3×3 nm is used.

Reflectance spectra are calculated by illuminating the system at normal incidence with x-polarized light using a plane wave source and recording the reflected power. To estimate the refractive index sensitivity of each structure, a series of reflectance spectra are calculated at different refractive indices of the media in the range n = 1.33–1.48. The induced spectral shift of the plasmon resonance peak is evaluated, and the sensitivity is calculated as the slope of the linear dependence of the peak position on the refractive index of the medium.

## 3. Results and Discussion

**Figure 2:**
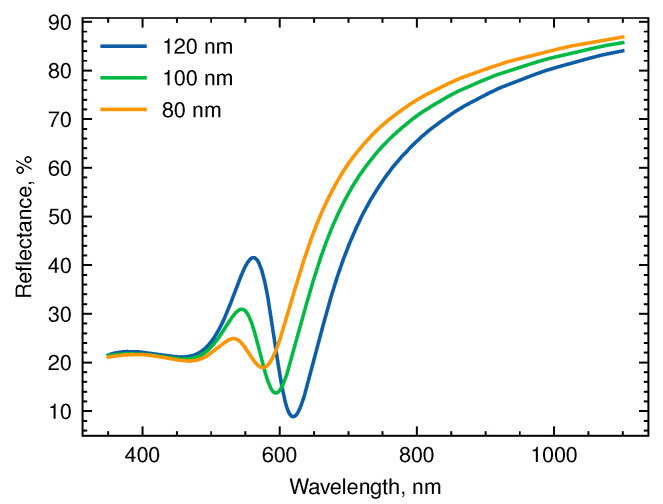
Reflectance spectra of Au-NHAs with different hole diameters at n = 1.33.

Figure 2 displays simulated reflectance spectra for single-layer Au NHAs with periodic structure and varying hole diameters of 80, 100, and 120 nm. These spectra exhibit a reflectance peak and dip that shift towards longer wavelengths as the hole diameter increases. Previous research suggests that the peak is attributed to a surface plasmon polariton within the nanohole layer, whereas the dip is linked to a localized surface plasmon resonance inside the holes [22,23]. Thus, the spectral position of the reflectance maximum is influenced by the distance between the holes, while the minimum is determined by the hole diameter.

In our model, the structure period (i.e., the distance between centers of the adjacent holes) scales with hole diameter as P=2D, which explains the observed simultaneous spectral shift of both the peak and the dip for systems with larger holes.

The addition of the perforated dielectric sublayer results in a small blue shift in resonance due to the lower effective refractive index of the medium compared with that of a substrate. The shift decreases with the thickness of the additional layer after 10–20 nm.

### 3.1. Effect of the Resonator Size

**Figure 3:**
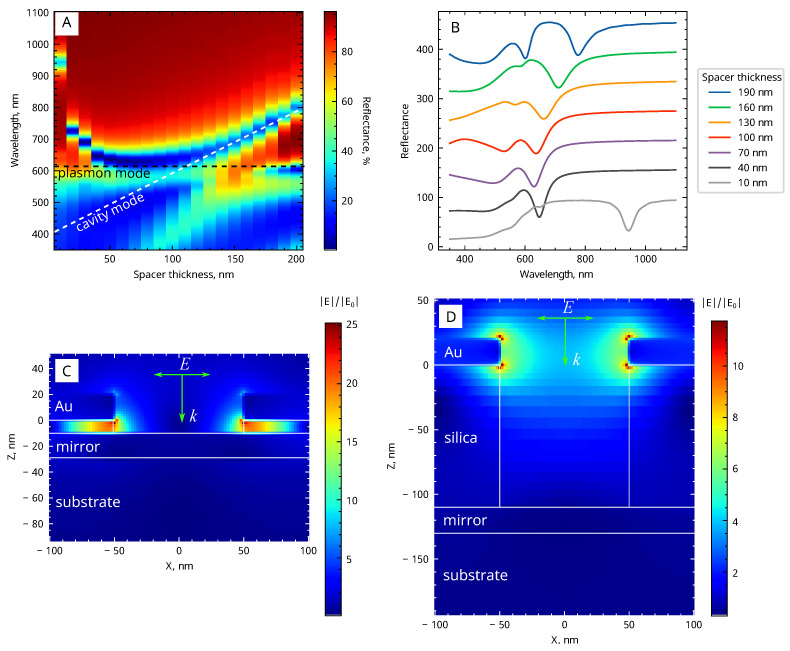
Near-field coupling of nanohole array LSPR with optical cavity modes in Au/SiO${}_{2}$/Au system with a diameter of 100 nm: (**A**) Reflectance spectra vs. spacer thickness as a 2D color map; (**B**) overlaid reflectance spectra for selected spacer thickness; (**C**,**D**) electric field distributions upon resonance at resonator lengths of 10 nm and 110 nm, respectively.

Optical properties of the system are significantly altered by the addition of a second layer of gold, which turns it into an optical resonator. The resonator length, which is the distance between the two gold layers, plays a crucial role in defining the coupling regimes that can be achieved. This is evident from the reflectance spectra of 100 nm gold NHA combined with a 20 nm thick gold film, presented in Figure 3A. A series of overlaid selected spectra is shown in Figure 3B. The spectra are stacked along the *y*-axis, allowing for direct visual comparison and observation of the spectral changes at different resonator lengths.

At low resonator length, a strong red shift of the reflection minimum is observed as the silica thickness decreases below 50 nm. This effect can be attributed to the electrostatic concept of image charges. Specifically, when a nanohole is situated near a metal layer, the instantaneous charges at the edges of the hole upon excitation of localized surface plasmon resonance induce charges of an opposite sign in the metal layer. This interaction is strongly distance-dependent and can result in a significant reduction of the resonance energy at small separation distances.

The effect is accompanied by a strong electric field enhancement, as shown in Figure 3C, which is important for high refractive index sensitivity of the system. The field enhancement factor $\left|E|/\right|E_{0}|$ observed at h=10 nm for Au/silica/Au NHA system with 100 nm holes exceeds 20. However, most of the enhanced near field is localized inside the silica layer, making it mostly inaccessible for the analyte.

When the distance between gold layers exceeds 50 nm, the effect of the electrostatic interaction becomes negligible. Consequently, varying the silica thickness from 50 to 100 nm has little effect on the resonance frequency, with the reflectance minimum situated near 620 nm, closely matching the position of undisturbed resonance in a single-layer gold NHA. Increasing the size of the resonator *h* causes a red shift of the Fabry-Pérot wavelength, with strong mode coupling occurring as it approaches the NHA plasmon resonance wavelength, causing anti-crossing of the resonances. This phenomenon is clearly seen in Figure 3A.

The comparison of the electric field plots, presented in Figure 3C,D suggests that the maximum electric field enhancement observed in the strong coupling regime is about $\left|E|/\right|E_{0}|$ = 10, which is approximately twice lower than that found for small silica thickness. However, the enhanced optical field is almost equally distributed between the top and bottom edges of the holes, thus being available for sensing the changes of refractive index.

**Figure 4:**
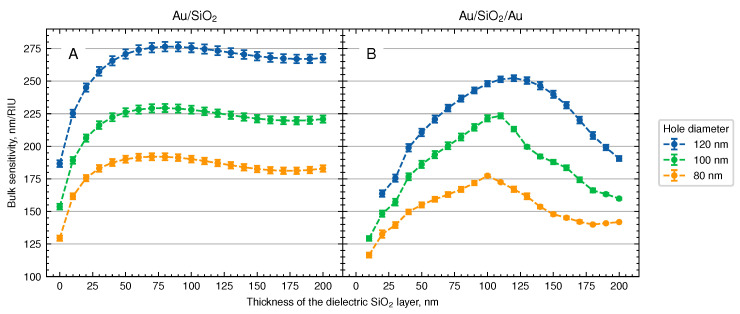
Effect of the thickness of the dielectric layer on the refractive index sensitivity of (**A**) elevated NHAs and (**B**) top-perforated Au/silica/Au system. Error bars show the standard error of sensitivity obtained from linear approximation of plasmon resonance versus refractive index.

To estimate the bulk refractive index sensitivity of a system, we examine the spectral shift of the long-wavelength reflectance minimum with changes in refractive index of the surrounding media for the two systems under study. The resulting data for NHAs of three different hole diameters and thicknesses of the silica layer are shown in Figure 4.

First, we consider the systems with a single Au layer, representing the ‘elevated nanoholes’, which demonstrated the high refractive index sensitivity earlier [14]. We observe that the sensitivity increases rapidly with the thickness of the silica layer (or the depth of the wells in the dielectric) up to about 50 nm before reaching a plateau, as shown in Figure 4A. This behavior is due to the exposure of the previously hidden enhanced electric field to the solution. The plateau is explained by the limited size of the enhanced electric field region.

In contrast, the sensitivity curves for Au/silica/Au systems shown in Figure 4B exhibit a different trend, with a maximum at a spacer thickness of 110–130 nm. The size of the optical cavity corresponding to the highest value of the refractive index sensitivity increases with the hole diameter: 100 nm for 80-nm nanoholes; 110 nm for 100-nm nanoholes; and 120 nm for 120-nm NHAs. Notably, the NHAs with larger hole diameters exhibit higher sensitivity.

The refractive index sensitivity of the coupled plasmonic-photonic system can be rationalized using an effective medium model that accounts for phase shifts. For constructive interference to occur, the total phase shift accumulated by the wave as it circulates in the cavity must equal 2Nπ (N=1,2,3…). The change in the refractive index of the solution leads to different phase shifts upon plasmon excitation in NHAs. Moreover, the effective refractive index of the cavity also depends on that of the analyte solution due to the nanoholes in the dielectric layer, which affects the phase shift in both cavity propagation and reflection. As a result, the overall phase shift changes and the resonance condition is no longer satisfied at the same wavelength.

The data obtained show that the Au/silica/Au system exhibits maximum sensitivity when the optical resonance of the coupled system is close to the plasmon resonance mode of the NHA. These conditions ensure the largest change of the NHA plasmon excitation phase shift [18].

In general, the Au/SiO${}_{2}$/Au structures demonstrate slightly lower bulk sensitivities compared with the corresponding Au/SiO${}_{2}$ structures with the same hole diameter. This effect has been observed in other systems upon coupling of plasmon modes to photonic cavity. For instance, the refractive index sensitivity of gold nanorods is found to decrease by a factor of 2.5 when a photonic cavity is introduced [18]. This phenomenon can be attributed to the increase in mode volume resulting from the presence of the cavity. However, our results suggest that under certain conditions the reduction of the sensitivity of the coupled photonic-plasmonic systems can be minimized. For instance, the sensitivity of 100-nm NHAs with a spacer thickness of 110 nm is approximately 225 nm/RIU for both systems.

### 3.2. Effect of the Inter-Hole Spacing

Another tuning parameter of the plasmonic NHA system is the inter-hole spacing, which affects the LSPR wavelength. Here, we study the effect of changing the inter-hole spacing on bulk refractive index sensitivity of the system.

Based on the results of the previous section, we use the fixed resonator distance of *h* = 110 nm, as it provides the highest sensitivity for the three studied systems. The inter-hole spacing is varied from 180 to 400 nm.

**Figure 5:**
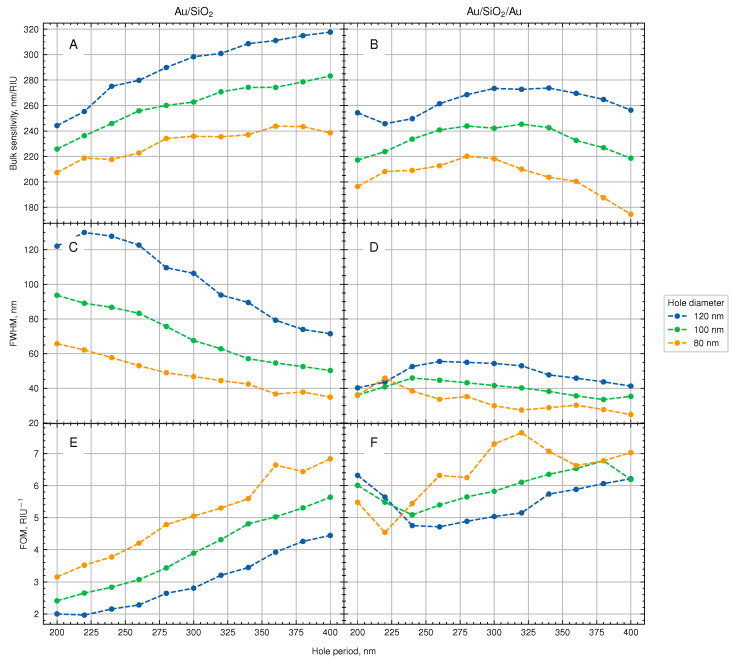
The effect of the inter-hole spacing on the sensing characteristics of Au/silica (**A**,**C**,**E**) and Au/silica/Au (**B**,**D**,**F**): bulk refractive index sensitivity (**A**,**B**); peak width at half maximum (**C**,**D**); and figure of merit (**E**,**F**).

As can be seen from the results presented in Figure 5B, there is an optimum range of inter-hole spacing for each hole diameter, providing higher refractive index sensitivity. The position of the maxima shifts to larger spacing values for larger hole diameters: 280, 300, and 320 nm for the NHAs with hole diameters of 80, 100, and 120 nm, respectively. The values of RI sensitivity reach 220, 240, and 275 RIU/nm for these systems. Thus, by tuning both the resonator size and the inter-hole spacing, the maximum RI sensitivity can be achieved.

The results obtained for the ‘elevated NHAs’ with a single gold film, presented in Figure 5A, show that the increase of the inter-hole spacing leads to even higher RI sensitivity. This, however, is due to red-shifting of the resonance peak with the NHA period.

At the same time, the coupling between plasmonic and photonic cavity modes results in a narrower resonance peak. The estimated values of the peak width at half maximum for the single-layer Au/SiO${}_{2}$ and the Au/SiO${}_{2}$/Au systems are presented in Figure 5C,D. The peak width of the former system is larger for short inter-hole distance and reaches 120 nm for the diameter of nanoholes of 120 nm and a period of 225 nm. For the photonic cavity system, the peak width does not change much with the period and its values are within the range of 40 ± 18 nm for the three studied hole diameters. As a result, the FOM values are higher for the cavity than for the single-layer NHA (see Figure 5E,F). For example, at a period P=300 nm, the *FOM* of the coupled photonic-plasmonic system is about 5–7 RIU${}^{-1}$ for different hole diameters, whereas for the single-layer NHAs, it lies within 3–5 RIU${}^{-1}$. It is worth mentioning that NHAs with smaller hole diameters demonstrate higher *FOM* values.

Higher *FOM* values are good for sensing, since they enable lower detection limits and higher sensitivity at the fixed wavelength $S^{*}$, defined by the change of intensity of the reflected light: $S^{*}=\Delta R/\Delta n$. Such modes of measurement are often more technologically relevant than the determination of the spectral position of the resonance peak.

The colloidal lithography approach could potentially be used to fabricate the gold-silica-gold NHAs discussed here. To create samples with a silica thickness smaller than the radius of nanospheres, a similar strategy to the one used for elevated NHAs (refer to reference [14] for more information) could be employed. This would involve vacuum deposition of a gold mirror layer, followed by the deposition of a colloidal mask and then vacuum deposition of the silica and gold layers. The desired structure would be achieved after removing the colloidal mask.

For samples with a larger distance between metal layers, similar steps could be followed, but with depositing both the gold and dielectric layers first, and then applying the colloidal mask and top gold layer. After lift-off, dry or wet chemical etching could be used to remove the dielectric material inside the holes.

## 4. Conclusions

In this study, we utilized the FDTD method to investigate the refractive index sensitivity of trilayer Au/silica/Au systems containing hexagonally arranged nanoholes in the top Au and silica layers. Our main goal was to compare the performance of these systems with the corresponding Au/silica NHAs and to evaluate how the spacing between the gold layers and the period of the nanohole array affect the system’s sensitivity and figure of merit.

Our results show that the introduction of a second metal layer creates a distance-dependent interaction between the plasmon resonance of the nanohole array and the photonic Fabry-Pérot modes. By adjusting the thickness of the dielectric spacer and the inter-hole spacing, we can significantly change sensitivity. The highest sensitivity is observed when the photonic cavity mode and NHA plasmon mode have close wavelengths.

Compared with “elevated NHAs”, we find that the presence of a cavity generally reduces the refractive index sensitivity of plasmonic NHAs. However, under certain conditions, this effect can be minimized. This, along with a decrease in resonance linewidth, leads to a noticeable increase in the figure of merit of the sensor. As a result, lower detection limits and enhanced sensitivity at a constant wavelength can be achieved. We believe that these findings have significant implications for understanding and improving the performance of plasmonic biosensors based on NHA topology.

## Data Availability

Data are contained within the article.

## Abbreviations

The following abbreviations are used in this manuscript:

NHA	Nanohole array
FDTD	Finite-difference time domain
*FOM*	Figure of merit
FWHM	Full width at half maximum
